# Physiological responses of broiler chickens fed reduced-energy diets supplemented with emulsifiers

**DOI:** 10.5713/ab.22.0142

**Published:** 2022-06-30

**Authors:** Elijah Ogola Oketch, Jung Woo Lee, Myunghwan Yu, Jun Seon Hong, Yu Bin Kim, Shan Randima Nawarathne, Josh Wen-Cheng Chiu, Jung Min Heo

**Affiliations:** 1 Department of Animal Science and Biotechnology, Chungnam National University, Daejeon 34134, Korea; 2 Ecolex Animal Nutrition, Kuala Lumpur 42920, Malaysia

**Keywords:** Broilers, Digestibility, Emulsifier, Performance, Reduced Energy, Tallow

## Abstract

**Objective:**

To investigate the physiological effects of exogenous emulsifiers in broiler chickens that were fed tallow-incorporated reduced-energy diets over 35 days.

**Methods:**

A total of 256 Ross 308 one-day-old broilers (42.28±0.16 g) were randomly allocated in a 2×2 factorial arrangement to 32 pens with eight chicks per cage. Birds were fed one of four dietary treatments as follows: i) positive control (PCN; energy sufficient diet); ii) negative control (NCN; energy-deficient diet, −100 ME kcal/kg); iii) PCL (PCN plus 0.05% emulsifier); and iv) NCL (NCN plus 0.05% emulsifier). Growth performance was evaluated weekly whereas assessments for the carcass traits, digestibility, some blood metabolites, ileal morphology, and meat quality were measured on d 21 and d 35.

**Results:**

Birds fed the NCL diet had higher (p<0.05) body weights, daily gains, daily feed intake, and improved feed efficiency over the entire 35-day period. Improvements (p<0.05) for the ileal digestibility of crude fat, energy, and dry matter commensurate with longer (p<0.05) villus heights were also observed with emulsifiers in the NCL and PCL diets. For the carcass measurements, only the liver weights were increased (p<0.05) with emulsifiers in the supplemented groups. For blood metabolites, higher (p<0.05) lipase levels were noticed with emulsifiers in the NCL and PCL diets. In addition, marginal reductions (p = 0.076; p = 0.095, respectively) were also noted with emulsifiers for the total cholesterol and triglyceride contents on d 35. Regarding meat quality, breast muscle yellowness was increased (p<0.05) with emulsifier use in supplemented groups.

**Conclusion:**

Our results suggest that emulsifier supplementation at 0.05% in diets could potentially improve the growth performance and nutrient digestibility of broilers over 35 days. This could compensate for the lower growth performance that could be recorded with fat-incorporated lower-energy diets.

## INTRODUCTION

Due to the price volatility of conventional feed ingredients, there is an ever-growing need to include relatively inexpensive alternatives such as fats in the diets. Such an approach might relatively reduce feed costs and simultaneously increase the energy density of the diets to meet the requirements of the modern fast-growing broilers. Fats are known to be a concentrated energy source, with at least twice as much energy as carbohydrates and proteins [1]. Alongside other important roles, supplemental fat use in diets could also slow down the digesta passage rate, thus allowing more time for nutrient digestion and absorption [1].

However, fat digestion and utilization is significantly more complex than those of other macronutrients and is largely dependent on the supply of adequate amounts of i) bile salts, ii) pancreatic lipase, and iii) co-lipase, a protein coenzyme required for optimal pancreatic lipase activity. It also involves several processes, including the breakdown of large fat droplets, emulsification, lipolysis, and mixed lipid-bile salt micelle formation before incorporation and reassembly into lipoproteins which are mainly chylomicrons. Chylomicrons are packed with triglycerides that will then be secreted through the portal system as portomicrons, this is the form in which fats are transported in the poultry [1].

Furthermore, there is an age-related depression in the capacity of newly hatched birds to utilize fats effectively, particularly animal fats. This could be because before 10 to 14 days of age, the gastrointestinal tract of young birds is incompletely or poorly developed [2,3]. Alongside inefficient bile salt recirculation, reduced levels of endogenous pancreatic lipase and fatty acid-binding proteins which are directly involved in the intracellular transport of fatty acids through the cytosol, have been reported to be low in young birds [1,2]. Thus, there is a consensus that the digestion and absorption of fats is physiologically limited in young birds.

Exogenous emulsifier use in poultry diets could mitigate these inadequacies, with reports of mostly improved growth performance and better fat utilization [4]. Emulsifiers act as molecular surfactants with both hydrophobic and hydrophilic properties that improves oil droplet distribution in oil-in-water emulsions. This process increases the active surface area for lipase to hydrolyse triglycerides into free fatty acids and monoacylglycerols, which are the absorbable units of fat [5]. Thus, fat digestion and absorption in the early stages of chick growth could be improved by exogenous emulsifier supplementation. Emulsifiers can be either natural or synthetic. Natural emulsifiers include bile salts, globin, and casein while synthetic surfactants could include calcium stearoyl-2-lactylate, glycerol distearate, and sodium stearoyl-2-lactylate [4].

Taken together, the use of supplemental fats (less than 4%) and exogenous emulsifiers in low-energy diets may potentially improve fat digestion and the energy efficiency of broiler diets. The overall growth performance could also be increased alongside the possibility of relatively reducing feed costs by incorporating more fats as inexpensive energy sources in diets [4]. Thus, this study tested the hypothesis that exogenous emulsifier addition into fat-infused reduced-energy diets has the potential to improve lipid digestion without compromising broiler performance. We conducted assessments for the growth performance, carcass traits, ileal digestibility, some blood metabolites, ileal histomorphology, and meat quality of broilers fed tallow-incorporated low-energy diets supplemented with emulsifiers over the course of 35 days.

## MATERIALS AND METHODS

The experimental protocol and procedures for the current study were reviewed and approved by the Animal Ethics Committee of Chungnam National University (Protocol Number; 202109A-CNU-113). The exogenous emulsifier product (Lipo AMP) was supplied by Ecolex Animal Nutrition, Kuala Lumpur, Malaysia. Lipo AMP is a multi-emulsifier system containing E481 (sodium stearoyl lactylate) and E471 (glycerol monostearate and glycerol distearate) emulsifiers. It also contains SiO_2_ as the anticaking agent.

### Birds and housing

A total of 256 Ross 308 broiler chicks (42.28±0.16 g) were raised over 35 days. Birds were weighed and allocated to their respective cages on arrival in a completely randomized design. Eight birds were used in each of the 32 raised wire floor pens (0.85×0.55×0.35 m^3^) and subjected to one of the four dietary treatments. Each pen was fitted with three nipple drinkers and a metal trough for the efficient provision of water and feed, respectively. Birds were allowed free access to fresh drinking water and feed throughout the entire experimental period. The birds were also vaccinated against infectious bronchitis and Newcastle disease at the hatchery. The environmental conditions were monitored regularly and adjusted accordingly as per the birds’ behaviour and age.

### Experimental design and diets

Birds were allocated to one of the four dietary treatments with 8 replicate pens per treatment in a completely randomized design. The diets were designed based on i) two different energy levels (positive control- PC, energy sufficient vs negative control- NC, energy sufficient [−100 ME kcal/kg]); and ii) with or without an exogenous emulsifier. The emulsifier was supplied at 0.05%. The dietary treatments were as follows: i) positive control (PCN; energy sufficient diet); ii) negative control (NCN; energy-deficient diet, −100 ME kcal/kg); iii) PCL (PCN plus 0.05% emulsifier); and iv) NCL (NCN plus 0.05% emulsifier). The diets were based on corn and soybean meal to meet the requirements of Ross 308 specifications [6].

Diets were fed over three phases starter (d 1 to 10), grower (d 11 to 21), and finisher (d 22 to 35). The starter diet was in a crumble form whereas both the grower and finisher diets were pelletized. Beef tallow was used as the fat source in all the experimental diets. Chromium (III) oxide (>99.9% purity, Sigma-Aldrich, St. Louis, MO, USA) was utilized as the indigestible marker for digestibility analysis in a proportion of 0.30% for all the treatments. Chromic oxide was preferred over other inert markers because it has a characteristic green colour that made it easier to confirm the addition of the marker in the diets. Feed samples were then collected and analysed for the gross energy, crude protein, and crude fat to verify proximity to the target values. The ingredients and the analysed nutrient compositions are recorded in Table 1.

### Growth performance measurements

Individual bodyweight measurements and feed consumption were then recorded weekly (days 7, 14, 21, 28, and 35). Further measurements were conducted on day 10 to enable growth performance evaluation for the starter period. From the feed consumed and body weights recorded, the average daily gain (ADG), mortality-corrected average daily feed intake (ADFI), and the feed conversion ratio (FCR) to depict the efficiency of converting feed to lean muscle was conducted. The pen was the experimental unit for growth performance evaluation.

### Post-mortem procedure and sample collection

Blood sample collections were carried out on d 21 and 35 of the experiment. One bird (closer to the mean body weight) was selected from each cage at a time. 6 birds per treatment were used for blood sample collection. The live body weights of selected birds were measured then the jugular vein was cut after which blood samples were collected into labelled vacutainer tubes for serum separation. The vacutainer tubes contained 10.8 mg spray-dried K2 ethylenediaminetetraacetic acid which works as an anticoagulant (Becton Dickinson, Franklin Lakes, NJ, USA). The collected blood samples were then transferred to the laboratory for serum separation.

Following the weight measurement and blood collection, the selected birds were then euthanized by cervical dislocation owing to the simplicity of the procedure [7]. The dead bird was then weighed minus the head and limb cut from the hock joint. This was recorded as the hot carcass weight. Abdominal incisions were then made to separate the ileum and jejunum from the gastrointestinal tract. The jejunum was defined as the part before the vitelline diverticulum (formerly Meckel’s diverticulum) whereas the ileum was defined as the part of the ileum extending from the vitelline diverticulum towards the ileo-caecal junction [3]. A cut was then made of the ileum (3 cm) from the vitelline diverticulum and then flushed with phosphate-buffered saline at pH 7.4. The sample was then placed in labelled containers containing 10% formaldehyde and stored for further ileal histomorphological measurements.

After the separation and collection of the ileal samples, the digesta of the ileal segment from birds subjected to the same treatment was gently flushed with distilled water as recommended by Ravindran et al [7] into labelled plastic containers. Thereafter, samples were then pooled from birds within a cage and frozen until further analysis. Thereafter, the liver, breast, and leg (thigh and drumstick) muscles were removed separately and weighed. The breast and leg samples that were weighed on day 35 were stored for meat quality analysis.

### Sample preparation and laboratory analysis

*Ileal histomorphological measurements*: Ring-shaped lengths of the ileal samples that were fixed in 10% formaldehyde were then excised, dehydrated, and embedded in paraffin wax as done by Yu et al [8]. Then transverse sections (5 μm) were cut, stained with haematoxylin-eosin then mounted on glass slides for viewing using an optical microscope (Olympus CX23; Olympus Corporation, Tokyo, Japan). The villus heights, base widths, and crypt of Lieberkühn depths were measured through the analysis of images of the histological sections from the software (NIS-Elements Viewer software, Version: 4.20; NIS Elements, Nikon, Melville, NY, USA). For all the ileal morphology measurements, cross-sections of 10 villi were randomly selected. The criterion for villus selection was based on the presence of a prominent lamina propria. Villus height and width, as well as crypt depth, were measured at 100× the objective magnification. All measurements taken from 10 villi per sample of an ileal segment were then expressed as the average for each bird. This was replicated for the 6 different transverse preparations per treatment and was analysed through statistical methods.

The villus height was defined as the distance from the villus tip to the base of the lamina propria whereas the villus width was measured at the halfway point of the villus height. The midway point was assumed to be representative and thus eliminated the need to measure the apical and basal width then dividing by 2 as done by Nain et al [9]. Additionally, the crypt depth was identified as the distance from the base of the villus to the muscularis mucosa. The ratio of the villus to crypt depth was estimated by dividing the villus height and mucosal crypt depth. Furthermore, because the villus is a cylindrical structure, the absorptive villus surface area (VSA) was calculated using the formulae: VSA = 2π×(average villus width/2)×villus height [9].

*Nutrient digestibility*: The apparent ileal digestibility (AID) of dry matter, gross energy, crude fat, and crude protein was determined to estimate the rate of nutrient disappearance at the terminal ileum. The previously collected digesta samples were thawed and dried at 55°C for 24 h, ground, and strained through a 0.75-mm sieve (ZM 200 Ultra-Centrifugal Mill; Retsch GmbH & Co., KG, Haan, Germany). The nutrient fractions were then analysed using standard procedures whereas the chromic oxide concentration was determined using the method of Fenton and Fenton [10]. The AID was then calculated as follows;


$$\text{Digestibility }(\%)=100-\left[100\times \left(\frac{{\text{M}}_{\text{diet}}\times {\text{N}}_{\text{digest}}}{{\text{M}}_{\text{digest}}\times {\text{N}}_{\text{diet}}}\right)\right]$$

M_diet_ is the marker concentration in the diet whereas N_digest_ is the nutrient concentration in ileal digesta whereas M_digest_ is the marker concentration in ileal digesta, and N_diet_ is the nutrient concentration in the diet.

*Blood parameters*: For plasma separation, the collected blood samples were centrifuged (LaboGene ScanSpeed 1248R; Gyrozen Co., LTD, Daejeon, Korea) at 3,000×g for 15 minutes at 4°C. After which, the obtained serum samples were separated into labelled 1.5 mL centrifuge tubes that were then preserved at −80°C (UniFreez U 400; DAIHAN Scientific Co., Ltd, Wonju, Korea) until further analysis. Total cholesterol, lipase, and triglyceride contents were then analysed using a Biochemistry Analyzer 7180 (HITACHI, Tokyo, Japan). The results were expressed in mg/dL.

*Meat quality assessments*: Regarding meat quality, the breast (*Pectoralis major*) and leg muscle samples were analysed for pH, colour, water holding capacity (WHC), and cooking loss. pH values were measured in duplicate using a pH meter (Mettler-Toledo GmbH, Sonnenbergstrasse, Schwerzenbach). For the meat colour, a colorimeter (Chroma Meter CR-410; Konica Minolta Ltd., Tokyo, Japan) was used to measure the CIE L* (lightness), CIE a* (redness, ±reddish green), and CIE b* (yellowness, ±yellowish blue). The cooking loss was determined as per the procedure of Kim and Chin [11] to gauge the percentage weight difference between fresh and cooked samples as compared to the weight of fresh muscle samples. Furthermore, the capacity of the muscle to retain bound water (WHC) was analysed by following the procedure of Jung et al [12].

### Statistical analyses

Collected data were analysed using the general linear model procedure for the two-way analysis of variance technique of IBM SPSS Statistics Windows, Version 26 (IBM Corp., Armonk, NY, USA) in a completely randomized design. The dietary energy levels and emulsifier supplementation at 0.05% were considered as the main effects. The pen was used as the experimental unit for the assessment of the growth performance parameters (BW, ADG, ADFI, and FCR). Selected birds that were euthanized for sample collection were considered as the experimental unit for carcass, blood parameters, ileal measurements, AID, and meat quality assessments. Statistical significance for the means was measured at p<0.05 and marginal effects (propensity for a significant effect) were measured at 0.05<p<0.10. When significance was noticed for the treatment effects, means were separated using Tukey’s multiple range test.

## RESULTS

### Growth performance

Body weights of birds fed the NCL diet (NC plus 0.05% emulsifier) improved (p<0.05) than that of other diet-fed birds on d 14, 21, 28, and 35 (p = 0.017, 0.016, 0.009, and 0.004, respectively; Table 2). Moreover, the ADG of NCL-fed birds were marginally higher than that of other diet-fed birds on d 7, 21, and 28 (p = 0.092, 0.064, and 0.081, respectively). Interestingly, ADG values were significantly improved in NCL-fed birds during the grower (d 11 to 21; p = 0.041) and finisher (d 22 to 35; p = 0.025) phases, and for the entire period (d 1 to 35; p = 0.002).

Furthermore, NCL-fed birds had a higher ADFI on d 21 only (p = 0.004) and during the growing period (p = 0.019) than other diet-fed birds. An interactive effect between the energy levels and emulsifier also influenced the ADFI in the NCL-fed birds on d 21 (p = 0.027). Concerning the FCR, lower values (p<0.05) that signify improved efficiency were noticed with the NCL-fed birds than with the other diet-fed birds on d 21 and 28 (p = 0.049 and 0.035, respectively) and during the entire period (d 1 to 35; p = 0.040).

### Carcass measurements

Emulsifier supplementation in both the NCL and PCL diets led to heavier liver weights (p<0.05) as at d 21 and 35 (Table 3). However, there were neither major effects (p>0.05) nor marginal effects (0.05<p<0.10) of emulsifier use on the carcass, breast muscle, and leg muscle percentages at d 21 and 35.

### Nutrient digestibility

The AID of crude fat at both d 21 and d 35 were (p<0.05) increased with emulsifiers in the NCL and the PCL (PC plus 0.05% emulsifier) diets relative to the non-supplemented NCN and PCN diets (Table 4). The AID of energy and dry matter were further improved (p<0.05) in NCL- and PCL-fed birds only at d 35. However, no notable results were observed for the AID of crude protein.

### Ileal histomorphological measurements

Higher (p<0.05) villus heights were observed with emulsifiers in the NCL- and PCL diets when compared to the non-supplemented PCN- and NCN-fed birds on d 21 (Table 5). Additionally, the VSA was marginally improved (p = 0.086) on d 21 with emulsifiers in the NCL and PCL diets.

### Blood metabolites

Blood lipase levels of PCL- and NCL-fed birds increased (p<0.05) more than those of other diet-fed birds on d 21 and 35 due to emulsifier addition (Table 6). Regarding the levels of total cholesterol and triglycerides, no major dietary effects of emulsifiers in diets were noticed on day 21. On day 35, emulsifier supplementation in the NCL and PCL diets led to marginal reductions (p = 0.076; p = 0.095, respectively) for the levels of total blood cholesterol and triglyceride levels.

### Meat quality

The leg and breast muscle colour (lightness, redness, and yellowness), pH, cooking loss, and WHC on d 35 are summarised in Table 7. Due to emulsifiers, the breast muscles of PCL- and NCL-fed birds indicated higher yellowness (p<0.05) than those of NCN- and PCN-fed birds. However, no significant dietary effects were observed for the leg muscles.

## DISCUSSION

The current study was conducted to determine the physiological effects of exogenous emulsifier supplementation in tallow-incorporated reduced-energy diets for broiler chicks. The performance benefits of supplying high energy in diets have been previously reported by Zhao and Kim [13]. Therefore, the relatively lower growth performance of the birds fed the negative control diet without emulsifiers, is not surprising. This observation could lead to a shift in the prioritization of energy for life preservation, as opposed to maintenance and muscle accretion; thus, growth performance was negatively affected. Upon emulsifier supplementation in the negative control diet, our results show that broilers fed a low-energy emulsifier-supplemented diet were more feed efficient, gained more weight, and consumed more feed per day, more so during the early stages (day 21). This could have led to the significantly higher body weights that were recorded for the supplemented birds in the current study and, has been reported elsewhere [3,14]. The improvements that were noticed with emulsifiers in the lower-energy diet (NCL) could be of importance for broiler production especially, under the limitation of energy provision using conventional sources such as corn.

The efficacy of emulsifiers in diets could vary depending on the hydrophilic–lipophilic balance (HLB), supplemental lipase use, and probably, the type and level of fats in the basal diets [3]. Thus, it should be noted that emulsifiers could also fail to elicit any significant improvements in growth performance and, thus has been reported [5]. Furthermore, this study also highlighted that the ability of emulsifiers to influence growth performance was related to the inclusion levels of fat in the diet. In the current study, the growth performance in birds fed the emulsifier-supplemented negative control diet with 1.5%, 2.5%, and 3.5% tallow for the starter, grower, and finisher phases, respectively, was better than that of birds fed the positive control diet with 2%, 3%, and 4% tallow for the starter, grower, and finisher phases, respectively.

The significant improvements that were noticed in the growth performance of birds with emulsifier-supplemented diets were consistent with the increases in nutrient digestibility. Our results are consistent with previous studies demonstrating that emulsifiers could improve fat digestibility [3,5]. Emulsifier effects on fat digestion are largely dependent on the extent of fat or water solubility in terms of the HLB. The HLB ranges from 0 to 20, with low HLB representing improved fat solubility in oil-in-water emulsions and high HLB signifying improved water solubility in water-in-oil emulsions. Based on Bancroft’s rule that an emulsifier should be soluble in the continuous (aqueous phase), a high HLB emulsifier is desirable because the small intestinal environment is predominantly aqueous, and birds are known to consume almost twice as much water as feed [4]. Considering the significant improvements in fat digestibility, emulsifiers could increase the active surface area for lipase to hydrolyse triglyceride molecules into fatty acids and monoglycerides, thus favouring the formation of mixed micelles. With efficient lipolysis and micelle formation, emulsifier use in diets could have a corrective effect on fat maldigestion and malabsorption that has been previously reported in young birds [5,15]. Beyond the ether extract, emulsifiers could also improve energy, dry matter, and protein digestibility and, thus has been reported [16–18].

The AID of energy, protein, and fat was the lowest in birds fed the negative control diet without the emulsifier. There is no coincidence that there could be deleterious effects on nutrient digestibility exerted by the higher levels of wheat in the non-supplemented negative control diet. Wheat bran was used to formulate the negative control diet; thus, the neutral detergent fibre levels in the NCN diet were much higher (Table 2). Wheat is high in water-soluble non-starch polysaccharides mainly arabinoxylans that could increase digesta viscosity [19]. With improved intestinal viscosity, the gut motility is also reduced with adverse impacts on the diffusion rate and convective transportation of emulsions, fatty acids, micelles, and lipase in the small intestine [20]. This could negatively influence the growth performance of mostly young birds [21] and nutrient digestibility especially fats and more so, saturated fats such as tallow which are the most affected by improved digesta viscosity among nutrients [22].

Considering the blood metabolites, the current study reported that blood lipase levels were significantly increased with emulsifier supplementation. Emulsifier use in broiler diets could lead to increased fat digestion and absorption as corroborated by the digestibility results from the current study. Improved fat digestibility could have the ripple effect of triggering the demand for more lipase production as suggested by Guerreiro Neto et al [5]. Furthermore, the lipase levels at d 21 were lower than those at d 35. This also suggests that there an age-related response that is independent of the substrate, for more lipase to be produced with an increase in age and, thus has been reported elsewhere [2]. With respect to the other blood metabolites that were analysed, the total cholesterol and triglyceride contents in the non-supplemented diets were higher due to tallow inclusion in the diets. In line with previous findings [23], emulsifier supplementation led to marginal reductions for the contents of total cholesterol and triglycerides.

The reduction effect of emulsifiers on the serum cholesterol and triglyceride concentrations could be due to a quick removal rate of chylomicrons (largely triglycerides) from blood, as suggested by Roy et al [16]. Furthermore, triglycerides can be hydrolysed by lipoprotein lipase (upon activation by apolipoprotein C-II) at the sn-1 and sn-3 positions, with the end products being two free fatty acids and sn-2- monoacylglycerols that will then be absorbed. The hypothesis of rapid chylomicron removal has been substantiated with reports of elevated lipoprotein lipase with emulsifier use in broiler diets [24]. Emulsifiers could also reduce cholesterol and triglyceride levels by stabilizing the phospholipid coating of portomicrons as suggested by Jones et al [25]. This could in turn decrease the release rate of cholesterol and triacylglycerols (sequestered in the portomicron interior) into the blood; hence, low levels can be observed. Further studies on the mechanism behind the effects of emulsifiers on blood metabolites should clear the inconsistencies noticed with elevated cholesterol levels with emulsifier supplementation also being reported in previous studies [14].

Furthermore, emulsifier supplementation had no significant effect on carcass measurements has it has been previously noted [3,5]. Due to variations in the efficacy of the different emulsifier efficacies, significant improvements in the breast and leg muscles due to emulsifiers in diets have also been reported elsewhere [18]. Considering the important role of the liver in avian fat metabolism, it should be noted that significantly improved liver weights were recorded for the emulsifier- fed birds. This could indicate a high lipid biosynthesis activity with emulsifier use, as suggested by Upadhaya et al [17]. The liver is not only the major lipogenesis site in birds, but it is also central to cholesterol conversion to bile salts, which are then reabsorbed enterohepatically. Additionally, the liver is involved in the clearance of portomicron remnants. Following the exogenous pathway, the previously mentioned action of lipoprotein lipase generates fatty acids from triglycerides (packed in portomicrons). The fatty acids are then transported to the adipose and muscle tissues via the capillaries, whereas the portomicron remnants, which are largely cholesterol and proteins (Apo A-I and apolipoprotein B), then go to the liver for apoE-mediated endocytosis. Future investigations could investigate the expression of apoproteins associated with chylomicrons assembly and lipid transport (apoE, apoB-48, apoC-II, apoC-III, and apoA-IV). To the best of our knowledge, only Bontempo et al [14] have investigated gene expression in the liver with emulsifiers till date. However, they reported no significant effect on Apo A-I and Apo B expression in the liver.

Ileal histomorphological measurements are important indicative biomarkers of gut health. Longer villus heights associated with increased cell mitosis could indicate an increased surface area for nutrient absorption; whereas deep crypts represent rapid tissue turnover, which increases the regenerative and proliferative activity in the intestinal mucosa [26]. It has been reported that emulsifiers have the potential to trigger epithelial changes that could improve gut health [27]. In this study, emulsifier supplementation significantly increased villus heights in the treated birds, with marginal improvements for the absorptive VSA. Similarly, Wickramasuriya et al [3] reported improved villus heights with emulsifier plus lipase supplementation in tallow-incorporated diets. The morphological improvements could result from the synergistic effects of emulsifier interactions with the other feed ingredients as opposed to the influence of the emulsifier in isolation [27]. However, variations could also exist with no significant effects of emulsifiers on gut morphology being previously reported [28].

To signify the exponential growth in the broiler sector, the growing period required to reach the desired market weight has greatly reduced over the years [29]. In contrast, the yield for preferable cut-up parts, such as breasts and thighs, has increased greatly. However, the rapid weight gain with high breast muscle yield, which is a genetic predisposition to modern broilers, has largely affected muscle structure and quality, increasing the frequency of abnormalities such as deep pectoral myopathy [30]. Therefore, assessments of meat quality indicators, such as appearance, pH, WHC, drip loss, and collagen content, are relevant because they impact the decisions made by consumers and processors, alike. Our results on muscle quality show that emulsifier use significantly increases breast muscle yellowness [7,16]. The observation could be because emulsifiers may be involved in the accumulation of lipid-soluble pigments responsible for yellowness, such as xanthophylls and, thus similar results have been reported elsewhere [3,14,17].

## CONCLUSION

Our results emphasize that 0.05% emulsifier use in diets could improve the growth performance and nutrient digestibility of broilers over 35 days. It may also influence fat metabolism (lipase, triglycerides, and cholesterol), intestinal morphology (villus heights and absorptive surface area), and breast muscle colour (yellowness). From a practical point of view, the study highlights the promising potential of improving broiler performance by incorporating the use of supplemental fats plus emulsifiers in reduced-energy diets.

## Figures and Tables

**Table 1:** Ingredients and analysed nutrient compositions of the experimental diets

Items	Positive control^1)^						Negative control^2)^					
	
	Starter		Grower		Finisher		Starter		Grower		Finisher	
	
	d 1 to 10		d 11 to 21		d 22 to 35		d 1 to 10		d 11 to 21		d 22 to 35	
	
	PCN	PCL	PCN	PCL	PCN	PCL	NCN	NCL	NCN	NCL	NCN	NCL
Ingredients (%)												
Corn	52.73	52.49	58.80	58.82	61.92	61.92	50.17	50.22	53.30	53.34	58.64	58.64
Wheat HRW^3)^	3.27	3.70	-	-	1.39	1.39	3.00	3.00	3.35	3.35	1.99	1.99
Wheat bran	-	-	-	-	-	-	4.26	4.16	4.11	4.02	4.13	4.05
Soybean meal 48%	36.78	36.54	33.85	33.77	28.70	28.70	35.85	35.85	32.37	32.37	27.75	27.78
Beef tallow	2.00	2.00	3.00	3.00	4.00	4.00	1.50	1.50	2.50	2.50	3.50	3.50
Limestone	1.80	1.80	1.22	1.22	1.00	1.00	1.80	1.80	1.22	1.22	1.00	1.00
Mono-calcium phosphate	1.80	1.80	1.40	1.40	1.30	1.30	1.80	1.80	1.40	1.40	1.30	1.30
Iodized salt	0.30	0.30	0.30	0.29	0.29	0.24	0.30	0.30	0.30	0.30	0.29	0.29
Vit-Min premix^4)^	0.30	0.30	0.30	0.30	0.30	0.30	0.30	0.30	0.30	0.30	0.30	0.30
Lysine-HCl	0.35	0.35	0.22	0.23	0.22	0.22	0.35	0.35	0.23	0.23	0.22	0.22
DL-Methionine	0.37	0.37	0.31	0.32	0.28	0.28	0.37	0.37	0.32	0.37	0.28	0.28
Chromic oxide^5)^	0.30	0.30	0.30	0.30	0.30	0.30	0.30	0.30	0.30	0.30	0.30	0.30
Pellet binder	-	-	0.30	0.30	0.30	0.30	-	-	0.30	0.30	0.30	0.30
Emulsifier product^6)^	-	0.05	-	0.05	-	0.05	-	0.05	-	0.05	-	0.05
Calculated analysis^7)^												
Metabolizable energy (kcal/kg)	3,000	3,000	3,100	3,100	3,200	3,200	2,900	2,900	3,000	3,000	3,100	3,100
Crude protein (%)	23.07	23.00	21.52	21.50	19.50	19.50	23.04	23.02	21.50	21.50	19.50	19.50
Crude fat (%)	2.10	2.10	2.30	2.30	2.30	2.30	2.20	2.20	2.30	2.30	2.40	2.40
Neutral detergent fibre (%)	8.78	8.79	8.66	8.65	8.69	8.69	10.30	10.26	10.27	10.23	10.20	10.16
Calcium (%)	1.20	1.20	0.90	0.90	0.78	0.78	1.21	1.21	0.90	0.90	0.79	0.79
Available phosphorous (%)	0.51	0.51	0.41	0.41	0.39	0.39	0.51	0.51	0.43	0.43	0.40	0.40
Total lysine (%)	1.47	1.47	1.29	1.30	1.16	1.16	1.47	1.47	1.29	1.28	1.15	1.15
Total methionine+cysteine (%)	1.09	1.09	1.00	1.00	0.91	0.91	1.10	1.10	1.00	1.01	0.91	0.92
Digestible lysine (%)	1.33	1.33	1.16	1.16	1.04	1.04	1.32	1.32	1.15	1.15	1.03	1.03
Digestible methionine+cysteine (%)	1.00	1.00	0.91	0.92	0.84	0.84	1.00	1.00	0.91	0.91	0.83	0.83
Analysed values^8)^												
Gross energy (kcal/kg)	3,522	3,529	3,762	3,795	4,075	4,052	3,439	3,415	3,699	3,670	3,922	3,964
Crude protein (%)	23.08	22.84	20.92	20.73	18.72	19.02	22.72	23.12	21.01	21.13	18.92	20.02
Crude fat (%)	2.11	2.15	2.24	2.32	2.45	2.35	2.41	2.37	2.36	2.16	2.47	2.31

1) PCN, positive control diet without the emulsifier; PCL, positive control diet with the emulsifier.

2) NCN, negative control diet without the emulsifier; NCL, negative control diet with the emulsifier.

3) Hard red winter wheat.

4) Provided per kilogram of diet: vitamin A, 12,000 IU; vitamin D_3_, 2,500 IU; vitamin E, 30 IU; vitamin K_3_, 3 mg; D-pantothenic acid, 15 mg; nicotinic acid, 40 mg; choline, 400 mg; and vitamin B_12_, 12 μg; Fe, 90 mg from iron sulphate; Cu, 8.8 mg from copper sulphate; Zn, 100 mg from zinc oxide; Mn, 54 mg from manganese oxide; I, 0.35 mg from potassium iodide; Se, 0.30 mg from sodium selenite.

5) Indigestible marker was sourced from Sigma-Aldrich, St. Louis, MO, USA.

6) Lipo AMP® emulsifier was supplied by Ecolex Animal Nutrition, Kula Lumpur, Malaysia.

7) The values were calculated according to the Ross 308 Broiler Nutrition Specifications (2019).

8) The values were obtained from chemical analysis of the feed samples according to standard procedures.

**Table 2:** Effects of dietary energy level and emulsifier supplementation in diets on the growth performance of broilers^1)^

Items	Positive control^2)^		Negative control^3)^		SEM^4)^	p-value		
		
	PCN	PCL	NCN	NCL		Energy^5)^	Emulsifier^6)^	E×E^7)^
Body weight (g)								
Day 1	42.48	42.40	42.36	42.37	0.163	0.662	0.460	0.438
Day 7	137.75	142.18	136.00	144.66	2.322	0.771	0.115	0.812
Day 14	282.71	295.67	279.07	301.78	3.458	0.824	0.017	0.515
Day 21	607.84	625.79	578.58	668.65	10.563	0.750	0.016	0.099
Day 28	1,118.46	1,166.73	1,069.95	1,222.41	17.997	0.921	0.009	0.159
Day 35	1,799.66	1,862.59	1,765.38	1,952.43	18.776	0.466	0.004	0.109
Average daily gain (g/d)								
Day 7	13.32	14.13	13.37	14.61	0.324	0.788	0.092	0.852
Day 14	20.99	21.92	20.52	22.44	0.532	0.979	0.191	0.646
Day 21	47.66	47.16	42.70	52.19	1.165	0.990	0.064	0.041
Day 28	72.95	77.28	70.20	79.11	1.831	0.901	0.081	0.537
Day 35	97.32	99.40	99.32	104.29	2.354	0.406	0.461	0.764
Day 1–10	14.58	15.29	14.46	15.26	0.295	0.906	0.212	0.936
Day 11–21	34.33	34.54	31.61	37.31	0.690	0.983	0.041	0.057
Day 22–35	85.13	88.35	84.77	91.70	1.069	0.489	0.025	0.392
Day 1–35	51.68	53.55	51.04	56.17	0.512	0.339	0.002	0.121
Average daily feed intake (g/d)								
Day 7	16.77	17.64	18.35	17.70	0.547	0.461	0.922	0.491
Day 14	28.37	28.22	28.29	28.39	0.495	0.965	0.986	0.904
Day 21	67.20	68.37	66.43	70.59	0.747	0.399	0.004	0.027
Day 28	104.98	106.31	106.33	109.35	0.663	0.109	0.111	0.532
Day 35	150.29	149.12	156.93	153.00	2.220	0.245	0.570	0.758
Day 1–10	19.36	19.38	19.70	18.62	0.598	0.869	0.661	0.650
Day 11–21	47.79	48.30	45.36	49.49	0.468	0.051	0.019	0.064
Day 22–35	127.63	127.81	131.63	131.18	1.171	0.123	0.938	0.909
Day 1–35	81.54	82.05	83.14	83.81	0.794	0.299	0.716	0.960
Feed conversion ratio (g/g)								
Day 7	1.27	1.25	1.38	1.23	0.050	0.676	0.382	0.498
Day 14	1.36	1.30	1.40	1.29	0.035	0.827	0.236	0.746
Day 21	1.44	1.45	1.50	1.38	0.040	0.945	0.044	0.361
Day 28	1.48	1.42	1.51	1.40	0.037	0.825	0.035	0.773
Day 35	1.57	1.51	1.58	1.50	0.045	0.984	0.385	0.820
Day 1–10	1.34	1.27	1.37	1.22	0.045	0.939	0.220	0.622
Day 11–21	1.40	1.37	1.45	1.33	0.028	0.930	0.212	0.398
Day 22–35	1.53	1.48	1.56	1.46	0.022	0.841	0.052	0.644
Day 1–35	1.58	1.53	1.59	1.50	0.022	0.865	0.040	0.352

1) Values are the mean of six replicates per treatment.

2) PCN, positive control diet without the emulsifier; PCL, positive control diet plus the emulsifier.

3) NCN, negative control diet without the emulsifier; NCL, negative control diet plus the emulsifier.

4) Pooled standard error of the mean.

5) Dietary energy level.

6) Emulsifier supplementation at 0.05%.

7) Dietary energy level×emulsifier supplementation.

**Table 3:** Effects of dietary energy levels and emulsifier supplementation in diets on the carcass traits and liver weights of broilers^1)^

Items	Positive control^2)^		Negative control^3)^		SEM^4)^	p-value		
		
	PCN	PCL	NCN	NCL		Energy^5)^	Emulsifier^6)^	E×E^7)^
Day 21								
Liver (g)	18.05	20.62	18.62	20.88	0.576	0.438	0.046	0.773
Breast (%)	21.44	21.89	21.63	20.49	0.374	0.425	0.648	0.291
Leg (%)	10.32	10.34	10.25	10.06	0.120	0.477	0.724	0.993
Carcass (%)	87.92	87.56	87.22	88.43	0.406	0.644	0.885	0.557
Day 35								
Liver (g)	56.17	61.33	56.50	61.00	1.144	0.994	0.047	0.886
Breast (%)	27.90	24.63	26.61	26.24	0.946	0.934	0.347	0.453
Leg (%)	10.84	10.89	10.67	10.60	0.096	0.102	0.439	0.766
Carcass (%)	91.67	92.03	91.98	91.72	0.297	0.998	0.931	0.609

1) Values are the mean of six replicates per treatment

2) PCN, positive control diet without the emulsifier; PCL, positive control diet plus the emulsifier.

3) NCN, negative control diet without the emulsifier; NCL, negative control diet plus the emulsifier.

4) Pooled standard error of the mean.

5) Dietary energy level.

6) Emulsifier supplementation at 0.05 %.

7) Dietary energy level×emulsifier supplementation.

**Table 4:** Effects of dietary energy levels and emulsifier supplementation in diets on the apparent ileal nutrient digestibility of broilers^1)^

Items	Positive control^2)^		Negative control^3)^		SEM^4)^	p-value		
		
	PCN	PCL	NCN	NCL		Energy^5)^	Emulsifier^6)^	E×E^7)^
Day 21 (%)								
Dry matter	63.16	64.29	63.59	64.10	0.528	0.911	0.453	0.774
Crude protein	78.24	80.47	78.65	78.68	0.522	0.523	0.297	0.312
Crude fat	77.24	80.02	77.21	79.77	0.432	0.872	0.009	0.901
Energy	61.91	61.96	61.00	62.50	0.635	0.920	0.579	0.553
Day 35								
Dry matter	72.39	76.76	75.76	76.60	0.512	0.143	0.026	0.110
Crude protein	79.81	79.37	78.76	79.36	0.352	0.947	0.433	0.989
Crude fat	79.18	81.43	78.82	82.50	0.261	0.512	0.008	0.195
Energy	79.59	80.84	78.94	81.14	0.396	0.875	0.044	0.578

1) Values are the mean of six replicates per treatment.

2) PCN, positive control diet without the emulsifier; PCL, positive control diet plus the emulsifier.

3) NCN, negative control diet without the emulsifier; NCL, negative control diet plus the emulsifier.

4) Pooled standard error of the mean.

5) Dietary energy level.

6) Emulsifier supplementation at 0.05%.

7) Dietary energy level×emulsifier supplementation.

**Table 5:** Effects of dietary energy levels and emulsifier supplementation in diets on the ileal histomorphology of broilers^1)^

Items	Positive control^2)^		Negative control^3)^		SEM^4)^	p-value		
		
	PCN	PCL	NCN	NCL		Energy^5)^	Emulsifier^6)^	E×E^7)^
Day 21								
Villus height (μm)	797.77	852.37	780.58	831.61	12.033	0.440	0.040	0.941
Crypt depth (μm)	101.43	104.95	105.45	108.47	1.427	0.202	0.266	0.932
Villus width (μm)	95.33	93.00	90.77	91.60	0.947	0.131	0.696	0.413
Villus height: crypt depth (μm)	7.95	8.25	7.41	7.62	0.124	0.230	0.308	0.858
Villus surface area (mm^2^)	0.24	0.25	0.22	0.24	0.004	0.125	0.086	0.843
Day 35								
Villus height (μm)	1,020.07	1,021.28	1,015.87	1,007.22	8.902	0.614	0.837	0.785
Crypt depth (μm)	98.92	99.35	101.65	100.11	1.575	0.587	0.861	0.759
Villus width (μm)	93.77	98.06	98.19	99.11	1.222	0.277	0.300	0.500
Villus height: crypt depth (μm)	10.31	10.36	10.00	10.11	0.149	0.365	0.800	0.917
Villus surface area (mm^2^)^8)^	0.30	0.32	0.31	0.32	0.005	0.553	0.354	0.447

1) Values are the mean of six replicates per treatment.

2) PCN, positive control diet without the emulsifier; PCL, positive control diet plus the emulsifier.

3) NCN, negative control diet without the emulsifier; NCL, negative control diet plus the emulsifier.

4) Pooled standard error of the mean.

5) Dietary energy level.

6) Emulsifier supplementation at 0.05%.

7) Dietary energy level×emulsifier supplementation.

8) Villus surface area = (2π×(average villus width/2)×villus height)/10^6^.

**Table 6:** Effects of dietary energy levels and emulsifier supplementation on the blood metabolites of broilers^1)^

Items	Positive control^2)^		Negative control^3)^		SEM^4)^	p-value		
		
	PCN	PCL	NCN	NCL		Energy^5)^	Emulsifier^6)^	E×E^7)^
Day 21 (mg/dL)								
Total cholesterol	146.30	124.65	127.13	117.80	6.537	0.339	0.359	0.646
Triglycerides	50.98	42.20	37.03	25.38	8.164	0.365	0.543	0.931
Lipase	10.30	12.65	10.47	11.08	0.243	0.175	0.010	0.101
Day 35 (mg/dL)								
Total cholesterol	134.30	113.87	126.73	104.73	5.497	0.456	0.076	0.951
Triglycerides	176.83	153.83	157.58	139.13	7.138	0.423	0.095	0.883
Lipase	12.38	14.72	12.47	14.63	0.331	1.000	0.005	0.882

1) Values are the mean of six replicates per treatment.

2) PCN, positive control diet without the emulsifier; PCL, positive control diet plus the emulsifier.

3) NCN, negative control diet without the emulsifier; NCL, negative control diet plus the emulsifier.

4) Pooled standard error of the mean.

5) Dietary energy level.

6) Emulsifier supplementation at 0.05%.

7) Dietary energy level×emulsifier supplementation.

**Table 7:** Effects of dietary energy levels and exogenous emulsifier supplementation in diets on the meat quality of broilers at d 35^1)^

Items	Positive control^2)^		Negative control^3)^		SEM^4)^	p-value		
		
	PCN	PCL	NCN	NCL		Energy^5)^	Emulsifier^6)^	E×E^7)^
Breast muscle								
Lightness (L*)	52.22	52.67	52.88	54.08	0.647	0.442	0.534	0.779
Redness (a*)	11.64	11.57	10.35	10.11	0.416	0.124	0.859	0.926
Yellowness (b*)	21.86	22.39	20.30	23.61	0.313	0.793	0.010	0.046
pH	5.93	5.90	5.98	5.86	0.021	0.862	0.100	0.306
Cooking loss (%)	17.35	17.45	17.44	17.47	0.895	0.991	0.940	0.983
Water holding capacity (%)	65.45	64.77	66.92	65.67	0.374	0.139	0.223	0.705
Leg muscle								
Lightness (L*)	53.64	54.21	51.30	52.12	0.774	0.178	0.661	0.939
Redness (a*)	11.70	11.62	10.72	9.77	0.463	0.153	0.592	0.649
Yellowness (b*)	20.46	21.71	20.59	21.83	0.587	0.950	0.292	0.968
pH	6.22	6.16	6.15	6.11	0.019	0.137	0.209	0.746
Cooking loss (%)	30.08	30.38	30.07	30.74	0.532	0.838	0.655	0.861
Water holding capacity (%)	75.87	73.97	74.70	74.48	0.784	0.867	0.513	0.602

1) Values are the mean of six replicates per treatment.

2) PCN, positive control diet without the emulsifier; PCL, positive control diet plus the emulsifier.

3) NCN, negative control diet without the emulsifier; NCL, negative control diet plus the emulsifier.

4) Pooled standard error of the mean.

5) Dietary energy level.

6) Emulsifier supplementation at 0.05%.

7) Dietary energy level×emulsifier supplementation.
